# Sexual Function and Depressive Symptoms in Young Women with Euthyroid Hashimoto’s Thyroiditis Receiving Vitamin D, Selenomethionine and Myo-Inositol: A Pilot Study

**DOI:** 10.3390/nu15122815

**Published:** 2023-06-20

**Authors:** Robert Krysiak, Karolina Kowalcze, Witold Szkróbka, Bogusław Okopień

**Affiliations:** 1 Department of Internal Medicine and Clinical Pharmacology, Medical University of Silesia, Medyków 18, 40-752 Katowice, Poland; wszkrobka@sum.edu.pl (W.S.); bokopien@sum.edu.pl (B.O.); 2 Department of Pediatrics in Bytom, School of Health Sciences in Katowice, Medical University of Silesia, Stefana Batorego 15, 41-902 Bytom, Poland; kkowalcze@sum.edu.pl

**Keywords:** inositol, mood, selenium, sexual functioning, thyroid autoimmunity, vitamin D

## Abstract

Thyroid autoimmunity is associated with an increased risk of sexual dysfunction. The aim of this study was to compare sexual functioning and depressive symptoms in women with Hashimoto’s thyroiditis receiving different treatments. The study included euthyroid women with autoimmune thyroiditis, untreated or receiving vitamin D, selenomethionine, or myo-inositol. Apart from measuring antibody titers and hormone levels, all participants completed questionnaires evaluating female sexual function (FSFI) and depressive symptoms (BDI-II). In untreated women, the overall FSFI scores and domain scores for desire, arousal, lubrication, and sexual satisfaction were lower than in women receiving vitamin D, selenomethionine, and myo-inositol. In the vitamin D-treated women, the total FSFI scores and scores for desire and arousal were higher than in women receiving the remaining micronutrients. The BDI-II score was lowest in the vitamin D-treated women and highest in the untreated patients with thyroiditis. Vitamin D-treated women were also characterized by lower antibody titers and higher testosterone levels than the women receiving the remaining micronutrients. There were no differences in sexual functioning and depressive symptoms between the selenomethionine- and myo-inositol-treated women. The study results suggest that although all antibody-lowering treatments are associated with better sexual functioning and well-being in young women with euthyroid autoimmune thyroiditis, the greatest benefits are observed in patients receiving vitamin D.

## 1. Introduction

Autoimmune thyroiditis, also referred to as Hashimoto’s thyroiditis, is the most frequent cause of hypothyroidism in developed countries, the most prevalent organ-specific autoimmune disorder, and one of the most common human disorders worldwide [1,2]. The disease is associated with cell-antibody-mediated cytotoxicity and apoptosis, leading to the progressive loss of follicular cells, the consequence of which is the replacement of thyroid parenchyma by lymphocytes and, at later stages, also by fibrous tissue [3]. The disorder is much more common in women than in men (a female-to-male ratio of 5–10:1) and is often diagnosed in the reproductive phase of life [1,2]. Despite the high prevalence, Hashimoto’s thyroiditis is still a poorly understood and understudied disorder of unknown pathogenesis, awaiting prevention strategies and new treatments. Surprisingly, little is known about the sexual functioning of women with this disorder. Moreover, most studies assessed the sexual functioning in individuals with thyroid hypofunction of different origins, and no separate analyses were conducted for individuals with thyroid autoimmunity. In the study by Oppo et al. [4], euthyroid women with Hashimoto’s thyroiditis were characterized by a decrease in the domain score for desire, but the remaining domains of female sexual functioning were less affected in comparison with the untreated hypothyroid women. Krysiak et al. [5] observed that the presence of euthyroid autoimmune thyroiditis was associated with a decrease in the Female Sexual Function Index (FSFI) score and impaired sexual desire, arousal, and sexual satisfaction. Deteriorating effects on women’s sexuality symptoms of thyroid autoimmunity and thyroid hypofunction were additive [5]. In the study by Bortun et al. [6], in women with autoimmune thyroiditis, the risk of female sexual dysfunction increased with the severity of thyroid hypofunction, and the most seriously disturbed were sexual desire, lubrication, and orgasm, followed by excitability and sexual satisfaction. Impaired sexual functioning in women with hypothyroidism correlated with its severity and the most pronounced disturbances were observed in individuals with overt thyroid hypofunction [7]. Women with hypothyroidism insufficiently treated with levothyroxine (the mean concentration of thyroid-stimulating hormone [TSH] equal to 4.4 mU/L) were characterized by an increased risk of sexual dysfunction [8]. Although thyroid hypofunction was associated with impaired desire, sexual dysfunction in these women occurred less frequently than in subjects with nodular goiter [9]. However, in the Chinese population, there were no significant differences between women with subclinical hypothyroidism and the control women with the prevalence of female sexual dysfunction and in the FSFI score [10]. Supplementation of exogenous thyroid hormones positively influenced sexual functioning. Levothyroxine treatment in hypothyroid women improved desire and orgasm [4]. Replacement of levothyroxine with levothyroxine/liothyronine combination therapy increased the domain scores for sexual desire and arousal and tended to increase the total FSFI scores, and these effects correlated with a treatment-induced increase in serum levels of free triiodothyronine and testosterone [11].

In contrast to autoimmune hypothyroidism, euthyroid Hashimoto’s thyroiditis does not require treatment with levothyroxine. In light of recent research, it seems that patients with euthyroid autoimmune thyroiditis may, however, benefit from treatment with vitamin D, selenium, and myo-inositol [12,13,14,15,16,17,18,19,20]. Circulating the levels of 25-hydroxyvitamin D correlated with TPOAb titers, duration of autoimmune thyroiditis, and thyroid volume [12]. Long-term administration of exogenous vitamin D decreased the thyroid antibody titers, not only in individuals with a low vitamin D status but also in subjects with 25-hydroxyvitamin D levels within the reference range [13,14]. Meta-analyses of observational studies showed that selenium treatment in patients with thyroid autoimmunity led to a reduction in titers of thyroid antibodies and the degree of thyroid hypoechogenicity, and its immunosuppressive properties were particularly pronounced in individuals receiving 200 µg of selenomethionine daily [15,16,17]. Finally, myo-inositol, the most abundant form of inositol [18], potentiated the impact of both vitamin D [19] and selenomethionine [20] on the thyroid antibody titers and slightly improved thyroid function.

The results of recent studies suggest the association between vitamin D status and female sexual response. A low vitamin D status correlated with impaired sexual function, and the degree of sexual dysfunction depended on the severity of vitamin D deficiency [21,22]. In comparison to the women with normal vitamin D status, women with vitamin D deficiency were characterized by a lower FSFI score and lower domain scores for desire, orgasm, and satisfaction, whereas for women with vitamin D insufficiency, it only reduced sexual desire [21]. In female patients with unexplained infertility, both vitamin D deficiency and vitamin D insufficiency were associated with lower values of the total FSFI score and all assessed domain scores, and similar differences were observed between women with vitamin D deficiency and vitamin D insufficiency [22]. Low 25-hydroxyvitamin D levels were associated with an increased prevalence of sexual dysfunction in pregnant women [23]. In young women with sexual dysfunction and 25-hydroxyvitamin D levels below 75 mmol/L, the intramuscular injection of high doses of cholecalciferol (300,000 IU at four-week intervals) increased their FSFI score [24]. In menopausal women, vitamin D administered with calcium, isoflavones, and inulin improved desire, orgasm, and pain, as well as the sexual domains of the Menopause-Specific Quality of Life Questionnaire [25]. Lastly, an improvement in sexual functioning was observed in postmenopausal women receiving calciferol vaginal suppositories [26]. The impact of vitamin D on sexual functioning has not been, however, investigated in individuals with thyroid disorders. Moreover, to the best of our knowledge, no study has assessed the relationships between female sexual response and selenium or inositol intake. To fill in the gap, the aim of the current study was to compare sexual functioning and depressive symptoms in women of reproductive age receiving three different non-hormonal thyroid antibody-lowering treatments.

## 2. Materials and Methods

The study was performed in accordance with the 1964 Helsinki Declaration, and the study protocol was approved by the local review board. All participants provided written informed consent after the investigator had explained the nature, significance, and implications of the study. Because the study did not prospectively assign individuals to a health-related intervention, trial registration was not applicable. The paper was prepared in accordance with the Enhancing the Quality and Transparency of Health Research (EQUATOR) Network guidelines for observational studies (STROBE).

### 2.1. Study Population

**Figure 1:**
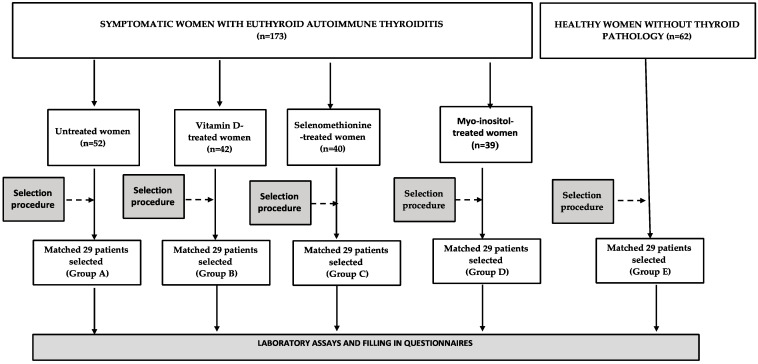
The flow of patients through the study.

The participants of this study were selected among young women (aged 18–45 years) with euthyroid Hashimoto’s thyroiditis, diagnosed six months earlier, and supervised by local healthcare providers cooperating with our research team. Women were eligible for the study if they fulfilled the following inclusion criteria: (a) plasma titers of thyroid peroxidase (TPOAb) and thyroglobulin (TgAb) antibodies above 100 U/mL; (b) ultrasound images suggestive of autoimmune thyroiditis (diffuse hypoechoic echogenicity, parenchymal heterogeneity, echogenic septations, and/or hypoechoic micronodularity); and (c) TSH and free thyroid hormone levels within the reference range (TSH between 0.4 and 4.5 mIU/L, free thyroxine between 10.2 and 21.0 pmol/L, and free triiodothyronine between 2.2 and 6.4 pmol/L). Depending on the treatment strategy during a six-month period between diagnosis and patient enrollment (the choice of which was determined by the patient’s preference and was unrelated to baseline vitamin D levels, selenium, and inositol homeostasis), the participants were categorized into one of the following four groups: untreated or receiving one of three treatment options (Group A), vitamin D preparations (100 µg [4000 IU] daily) (Group B), and selenomethionine (200 µg daily) or myo-inositol (2.0 g daily) (Group C). All these agents were taken orally, once daily in the morning over the entire period between diagnosis and enrollment. Dietary counseling in this period was provided by a certified nutritionist, and the daily intake of vitamin D, selenium, and inositol contained in food was assessed using an analysis of individual dietary questionnaires. The participants were selected from a larger number of eligible candidates (levothyroxine-naïve women), untreated, or receiving one of these treatment options based on a computer algorithm (Figure 1). The aim of this selection procedure was to create four study groups with similar titers of thyroid antibodies and similar levels of TSH and free thyroid hormones at the time of diagnosis. Women with autoimmune thyroiditis were compared with age-, body mass index-, and blood pressure-matched healthy women, serving as a control group (Group E). The number of individuals in each group (*n* = 29) exceeded the required one. An a priori sample size calculation showed that at least 25 patients per group must have been included to detect a 20% between-group difference in the total FSFI score (the primary endpoint) with a power of 0.8 and α of 5%. In order to limit the influence of seasonal confounds and seasonal fluctuations in the circulating levels of 25-hydroxyvitamin D and the remaining variables, similar numbers of individuals were assessed in each season, spring (*n* = 30), summer (*n* = 28), autumn (*n* = 29), and winter (*n* = 29).

The subjects were excluded if they met at least one of the following criteria: positive antibodies against thyrotropin receptor, other autoimmune or endocrine disorders, hepatic or renal failure, malabsorption syndrome, any other serious disorder, psychiatric problems, premature or early menopause, developmental or acquired anomalies of the female reproductive system, previous urogynecological operations that might affect sexual function, sexual inactivity, homosexual or bisexual orientation, pregnancy or lactation, and a body mass index above 35 kg/m^2^, as well as any pharmacological treatment (with the exception of exogenous vitamin D, selenomethionine, and myo-inositol).

### 2.2. Laboratory Assays

All laboratory assays were performed in duplicate to ensure the accuracy and reproducibility of the results by a person blinded to the patient’s identity and the study design and details. Venous blood samples were collected between 8:00 and 8:30 a.m. after 12 h of overnight fasting in a quiet and air-conditioned room (constant temperature of 23–24 °C). They were taken into the follicular phase after the patients had been resting in the seated position for a minimum of 30 min. Titers of TPOAb and TgAb, plasma levels of TSH, free thyroxine, free triiodothyronine, prolactin, estradiol, testosterone, sex hormone-binding globulin (SHBG), dehydroepiandrosterone sulfate (DHEA-S), and 25-hydroxyvitamin D were assayed by direct chemiluminescence using acridinium ester technology (ADVIA Centaur XP Immunoassay System, Siemens Healthcare Diagnostics, Munich, Germany). Concentrations of high-sensitivity C-reactive protein (hs-CRP) were measured using immunoassay with chemiluminescent detection (Immulite 2000XPi, Siemens Healthcare, Warsaw, Poland). The free androgen index (FAI) was calculated using multiplying testosterone levels (in nmol/L) by 100 and dividing by the SHBG levels (in nmol/L). The calculated parameters of thyroid homeostasis included the Jostel’s TSH index, the structure parameter inference approach (SPINA)-GT, as well as the SPINA-GD indices, which were calculated based on the TSH and free thyroid hormone levels using SPINA-Thyr 4.0.1 for Windows software. The Jostel’s index was calculated using the following formula: ln [thyrotropin] + 0.1345 × free thyroxine [27]. The SPINA-GT was assessed as follows: β_T_ × (D_T_ + thyrotropin) × (1 + K_41_ × standard concentration of thyroxine-binding globulin + K_42_ × standard concentration of transthyretin × free thyroxine)/(α_T_ × thyrotropin) [28,29]. SPINA-GD was calculated using the following equation: β_31_ × (K_M1_ + free thyroxine) (1 + K_30_ × standard concentration of thyroxine-binding globulin) × free triiodothyronine/(α_31_ × free thyroxine). The following constants were used in the equations: β_T_ = 1.1 × 10^−6^/s, D_T_ = 2.75 mU/L, K_41_ = 2 × 10^10^ L/mol, standard concentration of thyroxine-binding globulin = 300 nmol/L, K_T2_ = 2 × 10^8^ L/mol, standard concentration of transthyretin = 4.5 mmol/L, α_T_ = 0.1/L, β_31_ = 8 × 10^−6^/s, K_M1_ = 5 × 10^−7^ mol/L, K_30_ = 2 × 10^9^ L/mol and α_31_ = 0.026/L [28,29].

### 2.3. Questionnaires

Immediately after blood collection, all women considered for enrollment were asked to fill in three questionnaires assessing their sociodemographic characteristics, sexual functioning (FSFI), and depressive symptoms (the Beck Depression Inventory Second Edition: BDI-II). At the time of completing the questionnaires, neither the individuals nor the investigators were aware of the biochemical results. Although all potential participants filled in the questionnaires, only data from women selected for the study and data from control women were statistically analyzed.

The first questionnaire included questions concerning age, smoking, physical activity, education, occupational activity, profession, sexual partners, marital status, deliveries, miscarriages, and stress exposure, as well as obstetric and gynecological history.

The second questionnaire, the FSFI, is a 19-item self-report measure assessing women’s sexual functioning within the last four weeks across six domains: desire (questions 1 and 2; score range 1–5), arousal (questions 3–6; score range 0–5), lubrication (questions 7–10; score range 0–5), orgasm (questions 11–13; score range 0–5), satisfaction (questions 14–16; score range 1–5) and pain (questions 17–19; score range 0–5) [30]. The interpretation of the above partial scores is linear—the higher the score, the better sexual performance in each category. The overall score, being the sum of the scores for each item multiplied by a domain factor (0.6 for desire, 0.3 for arousal and lubrication, and 0.4 for orgasm, satisfaction, and pain), may range from 2 to 36. Sexual dysfunction was recognized when the total score is equal to or lower than 26.5 [31].

The last questionnaire, BDI-II, is a 21-item inventory that assesses specific cognitive, affective, and physical symptoms of depression in the last two weeks and their severity, corresponding well to a clinical diagnosis of depressive disorders outlined in the Diagnostic and Statistical Manual of Mental Disorders, Fourth Edition [32,33]. Each item consists of four alternative statements and the women were requested to endorse the one that best describes how they are currently feeling. Each answer was rated on a scale from 0 (absence of symptoms) to 3 (most severe symptoms). The total score, obtained by summing the scores across items, ranges from 0 to 63. A higher score on the BDI-II denotes more severe depression. The BDI-II scores of 0 to 13 denote no/minimal depression, scores of 14 to 19 denote mild depression, scores of 20 to 28 denote moderate depression, and scores of 29 to 63 denote severe depression [32].

### 2.4. Statistical Analysis

All raw data were logarithmically transformed to meet the assumption of equal variance. Between-group comparisons were analyzed using a one-way analysis of covariance followed by Bonferroni post hoc tests after consideration of age, smoking, body mass index, and blood pressure as potential confounders. For the categorical data (clinical characteristics of participants), the χ^2^ test was used. Bivariate relationships were analyzed using Pearson’s r tests (for two parametric variables); phi coefficient (for one parametric and one categorical variable); and point-biserial (for two categorical variables). Statistical significance was defined as a *p*-value corrected for multiple testing below 0.05.

## 3. Results

### 3.1. General Characteristics of the Study Groups

There were no differences between the study groups in age, body mass index, smoking habits, physical activity, education, occupational activity, type of work, the number of sexual partners, the number and duration of marriages, the number of deliveries and miscarriages, stress exposure, and blood pressure (both systolic and diastolic), as well as daily intake of vitamin D, selenium, and inositol (Table 1).

### 3.2. Biochemical Variables

At the time of diagnosis, there were no differences between the study groups in titers of TPOAb and TgAb, levels of TSH, free thyroxine and free triiodothyronine, and the calculated parameters of thyroid homeostasis (Table 2).

During the study, Groups A–D differed from Group E in TPOAb and TgAb titers and hs-CRP levels. The thyroid antibody titers and hs-CRP levels were lower in Groups B–D than in Group A. Moreover, the TPOAb titers and hs-CRP levels were lower in Group B than in Groups C and D. The TSH levels were higher in Group A than in Group E. There were differences in SPINA-GT between Group A and Groups B and E, as well as between Groups C and D and Group E. SPINA-GD was higher in Group C than in the remaining study groups. Testosterone levels and FAI were lower in Group A than in the remaining groups, as well as lower in Groups C and D than in Groups B and E. The levels of 25-hydroxyvitamin D were higher in Groups B and E than in the remaining groups. There were no differences in concentrations of 25-hydroxyvitamin D between Groups B and E, as well as between Groups A, C, and D. There were no between-groups differences in free thyroxine, free triiodothyronine, Jostel’s TSH index, prolactin, estradiol, SHBG, and DHEA-S (Table 3).

There were differences between Groups B–D and Group A in the percentage changes from baseline in thyroid antibody titers, TSH, and SPINA-GT during the six-month period between diagnosis and the study. Group B differed from Groups C and D in the percent changes from baseline in TPOAb titers. There were also differences in percentage changes from baseline in SPINA-GD between Group C and Groups A, B, and D (Table 4).

### 3.3. Sexual Functioning

Sexual dysfunction was diagnosed most frequently in Group A (48%), and most rarely in Group E (7%). Its frequency was greater in Groups A, C, and D than in the remaining two groups, and greater in Group A than in Groups C and D. The total FSFI score and domain scores for desire, arousal, lubrication, and sexual satisfaction were lower in Group A than in the remaining groups. Groups C and D differed from Group B in lower values of total FSFI score and in domain scores for desire and arousal. The domain score for sexual satisfaction was lower in Group B than in Group E. There were no differences in the total FSFI score and domain scores between Groups C and D (Table 5).

Sexual dysfunction was diagnosed most frequently in women with vitamin D deficiency (43%), and most rarely in individuals with a normal vitamin D status (14%). Women with vitamin D deficiency were characterized by a lower overall FSFI score, and by lower domain scores for desire, arousal, orgasm, and sexual satisfaction than women with a normal vitamin D status. The overall FSFI score and domain score for desire were higher in women with vitamin D insufficiency than in women with vitamin D deficiency, as well as lower in women with vitamin D insufficiency than in women with normal vitamin D status (Table 6).

### 3.4. Depressive Symptoms

The overall BDI-II score was higher in Group A than in the remaining groups, higher in Groups B–D than in Group E, and higher in Groups C and D than in Group B. The percentage of women with total and mild depressive symptoms was highest in Group A, while lowest in Group E (Table 7).

### 3.5. Correlations

The overall FSFI score inversely correlated with the BDI-II score (r values between −0.30 [*p* = 0.0392] and −0.46 [*p* = 0.0002]). The BDI-II score also correlated with scores for desire, arousal, lubrication, and sexual satisfaction (r values between −0.28 [*p* = 0.0491] and −0.44 [*p* = 0.0004]). Correlations with the BDI-II score were stronger for lubrication and sexual satisfaction (r values between −0.37 [*p* = 0.0128] and −0.46 [*p* = 0.0002]) than for desire and arousal (r values between −0.30 [*p* = 0.0392] and −0.36 [*p* = 0.0176]). There were positive correlations between the FSFI scores and domain scores for desire, arousal, lubrication and sexual satisfaction, and testosterone (r values between 0.28 [*p* = 0.0484] and 0.47 [*p* = 0.0002]) or FAI (r values between 0.31 [*p* = 0.0322] and 0.49 [*p* = 0.0001]). Correlations with testosterone and FAI were stronger for desire and arousal (r values between 0.38 [*p* = 0.0012] and 0.49 [*p* = 0.0001) than for lubrication and sexual satisfaction (r values between 0.28 [*p* = 0.0484] and 0.38 [*p* = 0.0014]). The overall FSFI score and domain scores for desire correlated with the circulating 25-hydroxyvitamin D levels and the mean daily vitamin D intake (all patients: r values between 0.38 [*p* = 0.0011] and 0.46 [*p* = 0.0002], Group A: r values between 0.35 [*p* = 0.0124] and 0.43 [*p* = 0.0005], Group B: r values between 0.39 [*p* = 0.0011] and 0.48 [*p* = 0.0001], Group C: r values between 0.30 [*p* = 0.0375] and 0.46 [*p* = 0.0002], Group D: r values between 0.29 [*p* = 0.0464] and 0.40 [*p* = 0.0008], Group E: r values between 0.34 [*p* = 0.0206] and 0.41 [*p* = 0.0008]). The BDI-II score positively correlated with antibody titers (TPOAb: r values between 0.39 [*p* = 0.0122] and 0.51 [*p* < 0.0001]; TgAb: r values between 0.34 [*p* = 0.0206] and 0.41 [*p* = 0.0008]) and hs-CRP (r values between 0.40 [*p* = 0.0006] and 0.47 [*p* = 0.0002]). Moreover, there were positive correlations between thyroid antibody titers and hs-CRP (r values between 0.43 [*p* = 0.0004] and 0.53 [*p* < 0.00001), as well inverse correlations between hs-CRP and the FSFI score and domain scores for libido, arousal, lubrication, and sexual satisfaction (r values between −0.29 [*p* = 0.0416] and −0.39 [*p* = 0.0008]). Lastly, in Group B, SPINA-GT inversely correlated with TPOAb titers (r = −0.40; *p* = 0.0006), TgAb titers (r = −0.32; *p* = 0.0224), and the BDI-II score (r = −0.28; *p* = 0.0462).

## 4. Discussion

### 4.1. Sexual Functioning in Untreated Euthyroid Women with Autoimmune Thyroiditis

In line with previous studies [4,5,6,9], women with euthyroid autoimmune thyroiditis were characterized by worse sexual functioning than their matched healthy peers. The affected women had impaired libido, arousal, lubrication, and sexual satisfaction. Sexual dysfunction in our patients was more pronounced than that reported by Oppo et al. (only hypolipidemia) [4] and in the study by Pasquali et al. (no differences in single domains of the FSFI questionnaire) [9]. However, the disorder impaired similar domains of sexual functioning to those described previously by our research group (desire, lubrication, and satisfaction) [5] and by Bortun et al. (desire, arousal, lubrication, and orgasm) [6]. The strength of our study was that, unlike previous reports, the included patients constituted a relatively homogenous population. Hashimoto’s thyroiditis was diagnosed on the basis of both markedly elevated titers of thyroid antibodies and a typical sonographic picture of the thyroid gland. Moreover, in all cases, the disorder was relatively recently diagnosed (six months earlier) and untreated with thyroid hormones. Differences in sexual functioning between the untreated patients and healthy subjects, as well as correlations between the FSFI and domain scores and hs-CRP levels, and between the thyroid antibody titers and hs-CRP levels, indicate that sexual dysfunction in euthyroid Hashimoto’s thyroiditis may be secondary to systemic inflammation induced by thyroid infiltration with inflammatory cells (mainly lymphocytes). Alternative explanations are not supported by our results. All study groups were characterized by similar sociodemographic characteristics. Moreover, at the time of completing the questionnaires, the participants (and the investigators) were unaware of the actual antibody titers and hormone levels, which minimized the subjectivity of answers in both questionnaires. The participants did not have overt comorbidities, as well as were not treated with other drugs. A multidirectional impairment of female sexual functioning associated with Hashimoto’s thyroiditis, reported in our study, suggests that all women with a sexual dysfunction of unknown origin should be assessed for the presence of autoimmune thyroid disease, even if TSH and free thyroid hormone levels are within normal limits.

### 4.2. Sexual Functioning in Euthyroid Women with Autoimmune Thyroiditis Receiving Micronutrients

A novel finding of the current study was that individuals receiving selenomethionine, an organically bound form of selenium with a greater absorption efficiency compared to inorganic forms of selenium [34], or myo-inositol, constituting about 99% of inositols contained in the intracellular pool of most tissues [35], were characterized by better sexual functioning than untreated women with this disorder. The beneficial effect in selenomethionine-treated patients is in line with the results of an animal study that showed that selenium nanoparticles increased the sexual behavior of Japanese quails [36]. However, though both agents were administered at doses found to reduce thyroid antibody titers [15,19], the overall FSFI score and domain scores for desire and arousal in women receiving these agents were lower than in the healthy controls. Interestingly, both global sexual functioning and individual domain scores did not differ between the individuals treated with selenomethionine and myo-inositol. Thus, our findings suggest that selenomethionine and myo-inositol administered at doses used in the current study are equipotent in improving sexual functions, but they are unable to completely normalize the female sexual cycle. Interestingly, selenomethionine treatment was associated with higher values of SPINA-GD, a parameter assessing the conversion of thyroxine to triiodothyronine [28,29], than the remaining treatments. Insufficient selenium intake, characterizing inhabitants of the Upper Silesia [37], may lead to a decrease in the activity of iodothyronine deiodinases, which are selenoproteins [38]. Thus, our finding probably reflects the improvement in the selenium status in this treatment group.

However, the most important finding of the present study was that the strongest effect on sexual function was observed in women receiving exogenous vitamin D. Calciferol treatment was associated with the improvement in all affected domains, as well as was superior to selenomethionine and myo-inositol treatment in the impact on desire and arousal. This finding cannot be explained by the baseline characteristics of the enrolled patients, which were similar in all treatment groups. Strict exclusion criteria minimized the impact of other disorders and the pharmacokinetic or pharmacodynamic interactions between agents assessed in the study and other drugs. Lastly, owing to the selection procedure, at the time of diagnosis, all groups of women with autoimmune thyroiditis were characterized by similar titers of TPOAb and TgAb and similar activity of the hypothalamic-pituitary-thyroid axis.

### 4.3. The Practical Significance of the Obtained Results

Based on the obtained results, some practical conclusions can be drawn. Firstly, the concomitant presence of sexual dysfunction is an argument in favor of the treatment with thyroid antibody-lowering agents, even if Hashimoto’s thyroiditis is not complicated by thyroid hypofunction. Secondly, considering the relationship between the baseline antibody titers and the improvement in sexual functioning, women with the highest titers of TPOAb and TgAb seem to benefit the most from the reduction in antibody titers. Thirdly, the degree of reduction in antibody titers (particularly in TPOAb) may serve as a predictor of the improvement in sexual functioning. Fourthly, vitamin D treatment seems to bring more benefits than selenium and inositol preparations to women with autoimmune thyroiditis complaining of sexual disturbances. Lastly, considering a stronger effect of vitamin D/selenomethionine [39] or vitamin D/myo-inositol [19] combination therapy than monotherapy on thyroid autoimmunity, euthyroid women with very high antibody titers and/or with more severe forms of sexual dysfunction may need to be treated either with both vitamin D and selenomethionine or with both vitamin D and myo-inositol.

### 4.4. The Putative Role of the Impact on Thyroid Function and Inflammation in Sexual Functioning of Micronutrient-Treated Euthyroid Women with Autoimmune Thyroiditis

In the current study, only a six-month vitamin D treatment normalized SPINA-GT, and only in this treatment group did SPINA-GT correlate with antibody titers. Because SPINA-GT estimates the maximum secretion rate of the thyroid gland under stimulated conditions [28,29], it seems that by reducing thyroid infiltration, vitamin D partially restores the function of preserved thyrocytes. However, the lack of differences in the levels of TSH and free thyroid hormones and the lack of correlations between SPINA-GT and sexual function does not allow us to conclude that the small improvement in thyroid secretory capacity plays a role in the improvement in sexual function. Moreover, our findings cannot be explained by the impact of the studied agents at the level of the pituitary thyrotropes because there were no between-group differences in Jostel’s TSH index, a marker of thyrotropic pituitary function [27].

The most probable explanation for the superiority of vitamin D over selenomethionine and myo-inositol is the strongest effect of calciferol on thyroid autoimmunity. In line with this explanation, the TPOAb titers were lower in calciferol-treated women than in women receiving selenomethionine or myo-inositol. Similar relationships, though not reaching the level of statistical significance, were observed in the case of TgAb. However, TgAb titers are regarded as a less sensitive and specific marker for thyroid autoimmune disorders than TPOAb titers [40], and this fact explains why between-group differences in the TgAb titers were less expressed than differences in the TPOAb titers. The beneficial effect of all treatment options on sexual functioning may be mediated by systemic inflammation. In line with this explanation, the levels of hs-CRP, an established marker of low-grade inflammation [41], were lower in women receiving vitamin D than the remaining two drugs, and correlated with both the antibody titers and domain scores for libido, arousal, lubrication, and sexual satisfaction. Moreover, rheumatoid arthritis [42] and lupus erythematosus [43], which are other disorders associated with systemic inflammation, were found to disturb all phases of the female sexual cycle.

### 4.5. The Putative Role of Vitamin D Status in Sexual Functioning of Micronutrient-Treated Euthyroid Women with Autoimmune Thyroiditis

In line with previous studies including patients without any concomitant disorders [21,22], sexual functioning of euthyroid women with Hashimoto’s thyroiditis depended on vitamin D status. This was best in women with a normal vitamin D status and worst in subjects with a vitamin D deficiency (defined as 25-hydroxyvitamin D levels below 50 nmol/L [20 ng/mL]). The difference was most pronounced for desire. Moreover, in the whole population and in all the study groups, the overall FSFI score and the domain scores for desire correlated with both the 25-hydroxyvitamin D levels and with mean daily vitamin D intake. These findings provide some interesting information about the role of vitamin D homeostasis in sexual functioning of women with thyroid autoimmunity. They indicate that Hashimoto’s thyroiditis women with 25-hydroxyvitamin D levels within the reference range and Hashimoto’s thyroiditis women with abnormally low 25-hydroxyvitamin D levels differ in the degree of sexual dysfunction. Thus, the assessment of the vitamin D status in all euthyroid women with autoimmune thyroiditis and impaired sexual functioning seems to be justified. Moreover, the obtained results suggest that the improvement in vitamin D status may contribute to a beneficial effect of calciferol treatment on libido and probably also on other aspects of the female sexual response. Furthermore, the highest 25-hydroxyvitamin D levels and the presence of only one individual with impaired vitamin D homeostasis may partially explain better sexual functioning in the group receiving exogenous calciferol preparations than in the remaining treatment groups. Lastly, the normalization of vitamin D status may be required for the optimization of sexual functioning also in females receiving selenomethionine or myo-inositol. Nevertheless, the obtained results cannot be regarded as a mere consequence of the between-group differences in vitamin D homeostasis. Despite normal 25-hydroxyvitamin D levels in almost all calciferol-treated women, the domain score for sexual satisfaction in this group of patients was lower than in healthy women. Moreover, correlations with the 25-hydroxyvitamin D levels and the mean daily vitamin D intake were limited to a global sexual functioning and libido. However, it should be kept in mind that the relationship between calciferol status and sexual functioning was not the primary endpoint, and therefore our study might have been underpowered to detect correlations with other aspects of female sexual response assessed by our research team.

### 4.6. The Putative Role of the Impact on Extra-Thyroid Hormones in Sexual Functioning of Micronutrient-Treated Euthyroid Women with Autoimmune Thyroiditis

Another possible explanation for our findings is the between-group differences in testosterone and FAI, a measure of the biologically active form of this hormone in the blood [44]. Both variables correlated with overall sexual functioning and impaired aspects of female sexual response assessed in the FSFI. Moreover, testosterone and FAI were higher in vitamin D-treated women than in women receiving selenomethionine or myo-inositol, as well as higher in selenomethionine- or myo-inositol-treated women than in untreated individuals with this disorder. The association with testosterone production is also supported by the fact that sexual drive and arousal, differing between women receiving calciferol and the remaining two treatment options, are regulated in women by testosterone to a greater degree than the remaining aspects of female sexual functioning [45,46]. The stimulating effect of calciferol on testosterone production and FAI is supported by previous observations. In healthy women, circulating 25-hydroxyvitamin D levels correlated with total testosterone and FAI [47]. Moreover, though no interventional studies were conducted in non-androgenic women, exogenous calciferol was found to increase testosterone levels in men with low-normal or subnormal testosterone concentrations, and this effect was mediated by vitamin D receptors in the Leydig cells [48]. Because the vitamin D receptor is present in theca cells [49], the impact of calciferol on ovarian androgen production may be similar to that observed in men. However, low testosterone cannot be regarded as the only mediator of sexual dysfunction because, despite no differences in testosterone and FAI between vitamin D-treated women and healthy women, both groups still differ in sexual satisfaction.

Unlike testosterone, the remaining extra-thyroid hormones do not seem to explain the obtained results. Increased prolactin production may contribute to the development and perpetuation of several autoimmune diseases, including Hashimoto’s thyroiditis [50], and may exert a negative impact on all aspects of female sexual functioning [51]. Moreover, thyroid autoimmunity was found to be associated with impaired function of the adrenal zona reticularis or increased metabolism of dehydroepiandrosterone [52,53], another hormone playing a role in the regulation of the female sexual response [45]. However, prolactin and DHEA-S, the best marker of zona reticularis activity (longer half-life and greater concentration in comparison with dehydroepiandrosterone, no circadian variation, and no changes in the menstrual cycle [46]), did not differ between the study groups, the mean levels of these hormones were within normal limits, and there were no correlations between prolactin and the DHEA-S levels and domain scores of FSFI. For similar reasons, impaired sexual functioning cannot be explained by the impact of estradiol. However, the role of prolactin excess cannot be completely ruled out in subjects with autoimmune hypothyroidism, not included in our study because of the association between thyroid hypofunction and increased prolactin production [54].

### 4.7. Depressive Symptoms in Euthyroid Women with Autoimmune Thyroiditis Receiving Micronutrients

The results of the current study support the association between autoimmune thyroiditis and the presence of depressive symptoms, observed previously by other authors in individuals with Hashimoto’s thyroiditis without thyroid hypofunction [55,56]. Although depressive symptoms were reported in 48% of untreated women with autoimmune thyroiditis, almost all of them these symptoms were mild, probably because of the normal levels of TSH and free thyroid hormones. In line with previous studies [5], depressive symptoms correlated with the degree of impairment in sexual function. Interestingly, these correlations were stronger for lubrication and sexual satisfaction than for libido and arousal. This finding, and only weak inverse correlations between lubrication and sexual satisfaction and testosterone and FAI, suggests that impairments of libido and arousal are probably more associated with decreased testosterone production, while lubrication and sexual satisfaction seem to be more related to depressive symptoms. Another important observation was a stronger effect of vitamin D on depressive symptoms in comparison with the remaining micronutrients, reflecting differences in the impact of these agents on the female sexual cycle and the severity of autoimmune thyroid destruction. The study design does not allow us, however, to conclude whether sexual dysfunction contributed to the increased number of patients with depressive symptoms and to their increased severity, or whether their impaired sexual functioning was a consequence of impaired mood. Thus, though previous studies showed that in women with depressive symptoms, calciferol, selenium, and inositol moderately improved mood [57,58,59], this study shows for the first time that this effect is also observed in women of reproductive age with euthyroid autoimmune thyroiditis, and suggests that the investigated micronutrients differ in the strength of mood-improving action. However, even the impact of vitamin D on depressive symptoms was moderate because individuals receiving calciferol were characterized by higher values of the BDI-II score than the control subjects without thyroid pathology, and were more frequently diagnosed with depressive symptoms. This means that calciferol cannot replace anti-depressive drugs, particularly in women with thyroiditis coexisting with more severe forms of depression. In addition to impaired sexual functioning, the between-group differences in the frequency and severity of depressive symptoms may be associated with differences in the impact of these micronutrients on thyroid autoimmunity and secretory capacity of the thyroid. In line with this explanation, the BDI-II score correlated with the antibody titers, hs-CRP, and vitamin D-treated women with SPINA-GT.

### 4.8. Study Limitations

The best study design to compare different treatment strategies would be to include euthyroid women with autoimmune thyroiditis, randomized to vitamin D, selenomethionine, myo-inositol, or placebo, with the assessment of sexual functioning and depressive symptoms before and after treatment. This design has not been used in the current study because of high costs, strict inclusion and exclusion criteria limiting the number of participants, and the lack of interest of many women in revealing information concerning one of the most intimate spheres of life. Because the study was non-randomized, the obtained results might have been influenced by latent confounders and selection bias. However, it should be underlined that the matched case-control study design used in the study has several advantages. Case-control studies are cost-efficient, may be carried out by small research teams, and are valuable pilot studies where little is known about the association between an intervention and an outcome [60]. Moreover, it was easier to encourage patients to fill in the questionnaire only once than on two occasions and to assure them that the questionnaire results will be revealed to their healthcare providers only if they agree.

The obtained results should be also interpreted in light of other study limitations. Owing to the small sample size, our findings should be interpreted as hypothesis-generating rather than definitive conclusions. Despite being well-validated, the utility of the FSFI and BDI-II questionnaires (as other self-report inventories) was limited by their subjectivity, and by the assessment at only one-time point (during, but not before, treatment). Because the study included only levothyroxine-naïve patients, the question of whether vitamin D, selenomethionine, and myo-inositol affect sexual functioning and mood in women with autoimmune thyroiditis, in whom TSH and free thyroid hormones within the reference range are a consequence of levothyroxine supplementation, requires further research. The impact on sexual functioning and depressive symptoms may be different in women with autoimmune hypothyroidism and in women after menopause, not participating in the current study. Although individuals with other overt endocrine disorders were excluded from the study, it cannot be completely ruled out that the presence of patients with preclinical stages of endocrine disorders (without specific symptoms), potentially causing sexual dysfunction and worsening mood, might have affected the obtained results. Despite an adequate iodine supply [61], the study population inhabited an area with low selenium intake [37], and it is not certain whether the impact of vitamin D, selenomethionine, and myo-inositol on sexual functioning and depressive symptoms is the same in patients with insufficient iodine intake and/or an adequate selenium status. Lastly, because the study does not provide insight into the molecular and cellular aspects of the association between vitamin D, selenomethionine, and myo-inositol and sexual functioning and mood, mechanisms underlying the obtained results require better understanding.

## 5. Conclusions

Untreated women with euthyroid autoimmune thyroiditis were characterized by a lower total FSFI score, as well as by lower domain scores for desire, arousal, lubrication, and sexual satisfaction in comparison with their healthy peers. The degree of sexual dysfunction depended on the antibody titers, plasma testosterone, and FAI, as well as correlated with the severity of depressive symptoms. Although all treatment options improved the sexual functioning and depressive symptoms, the strongest effect was observed in women receiving vitamin D. Our findings suggest that exogenous calciferol is superior to selenomethionine and myo-inositol in improving the sexual function and mood in women with autoimmune thyroiditis. Because of the limitations of the study design, our findings should be regarded as preliminary, and prospective, randomized, placebo-controlled studies with a larger sample size are required to confirm the obtained results. Further research is also warranted to better understand the biochemical mechanisms by which vitamin D, selenomethionine, and myo-inositol reduce thyroid antibody titers, and improve female sexual function and depressive symptoms.

## Figures and Tables

**Table 1:** General characteristics of the study population.

Variable	Group A	Group B	Group C	Group D	Group E
Number of patients	29	29	29	29	29
Age (years)	31 ± 7	32 ± 7	32 ± 6	31 ± 6	32 ± 7
Body mass index (kg/m^2^)	23.8 ± 4.2	23.4 ± 4.8	23.2 ± 4.0	24.0 ± 4.8	23.5 ± 4.3
Smokers (%)/Number of cigarettes a day (*n*)/Duration of smoking (months)	34/11 ± 7/82 ± 31	38/10 ± 8/84 ± 36	41/12 ± 8/88 ± 39	34/11 ± 6/80 ± 34	38/10 ± 7/86 ± 35
Physical activity: total/several times a week/once a week/once a month (%)	97/48/34/14	93/45/34/14	97/52/31/14	93/48/34/10	97/45/34/17
Primary or vocational/secondary/university education (%)	17/34/48	21/34/45	21/38/41	17/38/45	21/31/48
Occupational activity/Blue-collar/white-collar/pink-collar workers (%)	89/21/34/34	86/17/31/38	89/21/38/31	93/17/34/41	86/17/38/31
Number of sexual partners (*n*)	2.4 ± 1.2	2.4 ± 0.9	2.3 ± 0.8	2.2 ± 1.1	2.3 ± 1.0
Number of marriages (*n*)/duration of marriages (months)	1.2 ± 0.6/44 ± 18	1.3 ± 0.6/48 ± 17	1.2 ± 0.7/47 ± 19	1.1 ± 0.6/43 ± 20	1.3 ± 0.7/50 ± 20
Number of deliveries (*n*)/Number of miscarriages (*n*)	1.4 ± 0.7/0.6 ± 0.5	1.3 ± 0.6/0.7 ± 0.5	1.5 ± 0.8/0.5 ± 0.5	1.4 ± 0.6/0.6 ± 0.4	1.3 ± 0.7/0.4 ± 0.4
Stress exposure (%)	76	69	76	72	69
Systolic blood pressure (mm Hg)	124 ± 18	120 ± 16	121 ± 19	124 ± 17	120 ± 20
Diastolic blood pressure (mm Hg)	79 ± 6	77 ± 6	78 ± 6	78 ± 7	77 ± 6
Mean daily vitamin D intake ^1^ (µg)	8.9 ± 4.0	8.3 ± 3.0	9.1 ± 3.5	8.7 ± 3.8	9.5 ± 4.0
Mean daily selenium intake ^2^ (µg)	40 ± 10	38 ± 13	37 ± 12	38 ± 10	41 ± 11
Mean daily inositol intake ^3^ (mg)	750 ± 322	783 ± 352	812 ± 382	795 ± 320	822 ± 342

Group A: untreated euthyroid women with autoimmune thyroiditis. Group B: euthyroid women with autoimmune thyroiditis treated with vitamin D. Group C: euthyroid women with autoimmune thyroiditis treated with selenomethionine. Group D: euthyroid women with autoimmune thyroiditis treated with myo-inositol. Group E: healthy women (euthyroid women without thyroid autoimmunity). Unless otherwise stated, the data are presented as the mean ± standard deviation. ^1^ not including vitamin D tablets in Group C. ^2^ not including selenomethionine tablets in Group C. ^3^ not including myo-inositol tablets in Group D.

**Table 2:** Titers of thyroid antibodies and levels of TSH and free thyroid hormones in euthyroid women with autoimmune thyroiditis at the time of diagnosis.

Variable	Group A	Group B	Group C	Group D
TPOAb (IU/mL)	882 ± 324	912 ± 365	895 ± 279	928 ± 370
TgAb (IU/mL)	845 ± 382	832 ± 354	861 ± 324	870 ± 380
TSH (mIU/L)	3.0 ± 0.7	2.9 ± 0.8	3.1 ± 0.7	3.0 ± 0.6
Free thyroxine (pmol/L)	14.2 ± 2.1	14.5 ± 2.2	13.9 ± 2.0	14.3 ± 2.3
Free triiodothyronine (pmol/L)	3.0 ± 0.5	3.2 ± 0.6	3.1 ± 0.5	3.1 ± 0.6
Jostel’s TSH index	3.0 ± 0.2	3.1 ± 0.2	3.0 ± 0.2	3.0 ± 0.2
SPINA-GT (pmol/s)	2.07 ± 0.28	2.13 ± 0.35	1.99 ± 0.34	2.08 ± 0.29
SPINA-GD (nmol/s)	19.53 ± 2.59	20.41 ± 2.75	20.62 ± 3.11	20.04 ± 2.47

Group A: untreated euthyroid women with autoimmune thyroiditis. Group B: euthyroid women with autoimmune thyroiditis treated with vitamin D. Group C: euthyroid women with autoimmune thyroiditis treated with selenomethionine. Group D: euthyroid women with autoimmune thyroiditis treated with myo-inositol. The data are presented as the mean ± standard deviation. Reference values: TPOAb: <35 U/mL; TgAb: <35 U/mL; TSH: 0.4–4.5 mIU/L; free thyroxine: 10.2–21.0 pmol/L; free triiodothyronine: 2.2–6.4 pmol/L; Jostel’s TSH index: 1.3–4.1; SPINA-GT: 1.4–8.7 pmol/s; SPINA-GD: 20–60 nmol/s. Abbreviations: SPINA: structure parameter inference approach; TgAb: thyroglobulin antibodies; TPOAb: thyroid peroxidase antibodies; TSH: thyroid-stimulating hormone.

**Table 3:** Biochemical characteristics of the study population during the study.

Variable	Group A	Group B	Group C	Group D	Group E
TPOAb (IU/mL)	905 ± 348 ^&^	551 ± 290 *^#$&^	680 ± 221 *^&^	712 ± 286 *^&^	18 ± 8
TgAb (IU/mL)	882 ± 364 ^&^	535 ± 267 *^&^	623 ± 265 *^&^	640 ± 284 *^&^	17 ± 10
TSH (mIU/L)	3.1 ± 1.0 ^&^	2.6 ± 0.9	2.7 ± 0.8	2.7 ± 1.0	2.3 ± 0.8 *
Free thyroxine (pmol/L)	14.6 ± 2.8	15.8 ± 3.5	14.5 ± 2.9	14.8 ± 2.0	16.2 ± 3.7
Free triiodothyronine (pmol/L)	3.1 ± 0.7	3.5 ± 0.9	3.4 ± 0.8	3.2 ± 0.8	3.5 ± 0.9
Jostel’s TSH index	3.1 ± 0.2	3.1 ± 0.2	3.0 ± 0.2	3.0 ± 0.2	3.0 ± 0.2
SPINA-GT (pmol/s)	2.09 ± 0.41	2.47 ± 0.44 *	2.24 ± 0.47	2.27 ± 0.46	2.70 ± 0.49 *^#$^
SPINA-GD (nmol/s)	19.63 ± 2.83	20.38 ± 2.28	21.68 ± 2.56 *^#^&^	19.99 ± 2.44	19.98 ± 2.62
Prolactin (ng/mL)	17.2 ± 6.4	15.8 ± 8.2	16.3 ± 6.8	17.0 ± 7.0	16.5 ± 6.2
Estradiol (pmol/L)	432 ± 182	465 ± 150	415 ± 186	480 ± 180	446 ± 167
Testosterone (pmol/L)	1.02 ± 0.32	1.61 ± 0.38 *^#$^	1.25 ± 0.40 *	1.30 ± 0.34 *	1.65 ± 0.41 *^#$^
SHBG (nmol/L)	57 ± 16	64 ± 14	58 ± 17	61 ± 15	65 ± 21
FAI (%)	1.79 ± 0.42	2.51 ± 0.48 *^#$^	2.15 ± 0.50 *	2.13 ± 0.46 *	2.53 ± 0.43 *^#$^
DHEA-S (μmol/L)	6.4 ± 2.5	7.0 ± 2.5	6.8 ± 2.1	6.5 ± 2.5	7.2 ± 2.2
25-hydroxyvitamin D (nmol/L)	64 ± 28	106 ± 28 *^#$^	70 ± 26	66 ± 20	92 ± 32 *^#$^
hs-CRP (mg/L)	3.5 ± 1.2 ^&^	2.0 ± 0.6 *^#$&^	2.6 ± 0.9 *^&^	2.7 ± 0.8 *^&^	1.2 ± 0.3

Group A: untreated euthyroid women with autoimmune thyroiditis. Group B: euthyroid women with autoimmune thyroiditis treated with vitamin D. Group C: euthyroid women with autoimmune thyroiditis treated with selenomethionine. Group D: euthyroid women with autoimmune thyroiditis treated with myo-inositol. Group E: healthy women (euthyroid women without thyroid autoimmunity). The data are presented as the mean ± standard deviation. * *p* < 0.05 vs. group A. ^^^ *p* < 0.05 vs. Group C. ^#^ *p* < 0.05 vs. Group C. ^$^ *p* < 0.05 vs. Group D. ^&^ *p* < 0.05 vs. Group E. Reference values for young women in the follicular phase: TPOAb: <35 U/mL; TgAb: <35 U/mL; TSH: 0.4–4.5 mIU/L; free thyroxine: 10.2–21.0 pmol/L; free triiodothyronine: 2.2–6.4 pmol/L; Jostel’s TSH index: 1.3–4.1; SPINA-GT: 1.4–8.7 pmol/s; SPINA-GD: 20–60 nmol/s; prolactin: 5.0–29.0 ng/mL; estradiol: 220–650 pmol/L; testosterone: 0.4–2.1 nmol/L; SHBG: 30–140 nmol/L; FAI: <5%; DHEA-S: 2.0–11.0 μmol/L; androstenedione: 1.2–8.0 nmol/L; 25-hydroxyvitamin D: 75–150 nmol/L. Abbreviations: DHEA-S: dehydroepiandrosterone-sulfate; FAI: free androgen index; hs-CRP: high-sensitivity C-reactive protein; SHBG: sex hormone-binding globulin; SPINA: structure parameter inference approach; TgAb: thyroglobulin antibodies; TPOAb: thyroid peroxidase antibodies; TSH: thyroid-stimulating hormone.

**Table 4:** Percentage changes in titers of thyroid antibodies and levels of TSH during the six-month period of diagnosis and during the study.

Variable	Group A	Group B	Group C	Group D
Δ TPOAb	3 ± 8	−40 ± 18 *^#$^	−24 ± 14 *	−23 ± 12 *
Δ TgAb	4 ± 8	−36 ± 24 *	−28 ± 14 *	−26 ± 14 *
Δ TSH	3 ± 6	−10 ± 8 *	−13 ± 10 *	−9 ± 8 *
Δ Free thyroxine	3 ± 8	9 ± 15	4 ± 10	3 ± 8
Δ Free triiodothyronine	3 ± 10	9 ± 14	10 ± 16	3 ± 11
Δ Jostel’s TSH index	3 ± 11	0 ± 8	0 ± 7	0 ± 12
Δ SPINA-GT	1 ± 6	16 ± 16 *	12 ± 11 *	9 ± 10 *
Δ SPINA-GD	1 ± 4	0 ± 3	5 ± 5 *^^$^	0 ± 4

Group A: untreated euthyroid women with autoimmune thyroiditis. Group B: euthyroid women with autoimmune thyroiditis treated with vitamin D. Group C: euthyroid women with autoimmune thyroiditis treated with selenomethionine. Group D: euthyroid women with autoimmune thyroiditis treated with myo-inositol. The data are presented as the mean ± standard deviation. * *p* < 0.05 vs. Group A. ^^^ *p* < 0.05 vs. Group B. ^#^ *p* < 0.05 vs. Group C. ^$^ *p* < 0.05 vs. Group D. Abbreviations: SPINA: structure parameter inference approach; TgAb: thyroglobulin antibodies; TPOAb: thyroid peroxidase antibodies; TSH: thyroid-stimulating hormone.

**Table 5:** Sexual functioning in the study population.

Variable	Group A	Group B	Group C	Group D	Group E
FSFI score	26.95 ± 3.10 ^&^	31.40 ± 2.25 *^#$^	30.02 ± 2.18 *^&^	29.59 ± 3.02 *^&^	32.14 ± 2.53
FSFI score ≤ 26.55 (*n*%)	14 (48) ^&^	3 (10) *^#$^	7 (24) *^&^	8 (28) *^&^	2 (7)
Sexual desire	3.80 ± 0.90 ^&^	5.15 ± 0.58 *^#$^	4.61 ± 0.76 *^&^	4.48 ± 0.82 *^&^	5.39 ± 0.60
Sexual arousal	4.05 ± 0.85 ^&^	5.10 ± 0.82 *^#$^	4.67 ± 0.53 *^&^	4.51 ± 0.62 *^&^	5.28 ± 0.65
Lubrication	4.45 ± 0.84 ^&^	5.22 ± 0.65 *	5.12 ± 0.48 *	4.95 ± 0.78 *	5.24 ± 0.50 *
Orgasm	5.15 ± 0.75	5.40 ± 0.37	5.24 ± 0.51	5.20 ± 0.65	5.42 ± 0.46
Sexual satisfaction	4.40 ± 0.72 ^&^	5.18 ± 0.68 *^&^	5.20 ± 0.51 *^&^	5.23 ± 0.56 *^&^	5.50 ± 0.38
Pain	5.10 ± 0.68	5.35 ± 0.42	5.18 ± 0.58	5.22 ± 0.55	5.31 ± 0.45

Group A: untreated euthyroid women with autoimmune thyroiditis. Group B: euthyroid women with autoimmune thyroiditis treated with vitamin D. Group C: euthyroid women with autoimmune thyroiditis treated with selenomethionine. Group D: euthyroid women with autoimmune thyroiditis treated with myo-inositol. Group E: healthy women (euthyroid women without thyroid autoimmunity). Unless otherwise stated, the data are presented as the mean ± standard deviation. * *p* < 0.05 vs. Group A. ^#^ *p* < 0.05 vs. Group C. ^$^ *p* < 0.05 vs. Group D. ^&^ *p* < 0.05 vs. Group E. Abbreviations: FSFI: Female Sexual Function Index.

**Table 6:** Sexual functioning in patients with different vitamin D status.

Variable	Vitamin D Deficiency	Vitamin D Insufficiency	Normal Vitamin D Status
Number of patients: total/Group A/Group B/Group C/Group D/Group E (*n*)	21/8/0/6/7/0	53/14/1/13/13/12	71/7/28/10/9/17
FSFI score	28.32 ± 3.20 *^#^	29.58 ± 2.35 ^#^	30.84 ± 2.84
FSFI score ≤ 26.55 (*n* %)	9 (43) *^#^	15 (28) ^#^	10 (14)
Sexual desire	4.02 ± 0.98 *^#^	4.50 ± 0.84 ^#^	5.03 ± 0.78
Sexual arousal	4.41 ± 0.92 ^#^	4.60 ± 0.85	4.90 ± 0.88
Lubrication	4.89 ± 0.84	4.93 ± 0.78	5.09 ± 0.58
Orgasm	5.01 ± 0.65 ^#^	5.28 ± 0.58	5.35 ± 0.46
Sexual satisfaction	4.84 ± 0.75 ^#^	5.08 ± 0.65	5.19 ± 0.60
Pain	5.15 ± 0.53	5.19 ± 0.55	5.28 ± 0.48

Vitamin D deficiency was defined as plasma 25-hydroxyvitamin D levels below 50 nmol/L (20 ng/mL); vitamin D insufficiency was defined as plasma 25-hydroxyvitamin D levels between 50 and 75 nmol/L (20 and 30 ng/mL), normal vitamin D status was defined as plasma 25-hydroxyvitamin D levels between 75 and 150 nmol/L (30 and 60 ng/mL). The data are presented as the mean ± standard deviation. * *p* < 0.05 vs. vitamin D insufficiency. ^#^ *p* < 0.05 vs. normal vitamin D status. Abbreviations: FSFI: Female Sexual Function Index.

**Table 7:** Depressive symptoms in the study population.

Variable	Group A	Group B	Group C	Group D	Group E
BDI-II score	12.9 ± 3.5 ^&^	9.2 ± 3.0 *^#$&^	11.2 ± 2.9 *^&^	11.0 ± 3.4 *^&^	7.3 ± 2.8
Depression symptoms (*n*%)	14 (48) ^&^	6 (21) *^#$&^	10 (34) *^&^	10 (34) *^&^	4 (10)
Mild symptoms (*n*%)	13 (45) ^&^	6 (21) *^#$&^	10 (34) *^&^	10 (34) *^&^	4 (10)
Moderate symptoms (*n*%)	1 (3)	0 (0)	0 (0)	0 (0)	0 (0)
Severe symptoms (*n*%)	0 (0)	0 (0)	0 (0)	0 (0)	0 (0)

Group A: untreated euthyroid women with autoimmune thyroiditis. Group B: euthyroid women with autoimmune thyroiditis treated with vitamin D. Group C: euthyroid women with autoimmune thyroiditis treated with selenomethionine. Group D: euthyroid women with autoimmune thyroiditis treated with myo-inositol. Group E: healthy women (euthyroid women without thyroid autoimmunity). Unless otherwise stated, the data are presented as the mean ± standard deviation. * *p* < 0.05 vs. Group A. ^#^ *p* < 0.05 vs. Group C. ^$^ *p* < 0.05 vs. Group D. ^&^ *p* < 0.05 vs. Group E. Abbreviations: BDI-II: Beck Depression Inventory-Second Edition.

## Data Availability

The data that support the findings of this study are available from the corresponding author upon reasonable request.

## Abbreviations

BDI-II	Beck Depression Inventory-Second Edition
DHEA-S	dehydroepiandrosterone-sulfate
FAI	free androgen index
FSFI	Female Sexual Function Index
SHBG	sex hormone-binding globulin
SPINA	structure parameter inference approach
TgAb	thyroglobulin antibodies
TPOAb	thyroid peroxidase antibodies
TSH	thyroid-stimulating hormone
